# Tracheal Repair with Human Umbilical Cord Mesenchymal Stem Cells Differentiated in Chondrocytes Grown on an Acellular Amniotic Membrane: A Pre-Clinical Approach

**DOI:** 10.3390/life11090879

**Published:** 2021-08-26

**Authors:** Paulo Ricardo Baggio Simeoni, Rossana Baggio Simeoni, Paulo André Bispo Machado Júnior, Meila Bastos de Almeida, Dilcele Silva Moreira Dziedzic, Nádia Nascimento da Rosa, Priscila E. Ferreira Stricker, Anna Flávia Ribeiro dos Santos Miggiolaro, Guilherme Naves, Nelson Bergonse Neto, Lucia de Noronha, Julio Cesar Francisco, Katherine Athayde Teixeira de Carvalho, Luiz Cesar Guarita-Souza

**Affiliations:** 1 Experimental Laboratory of Institute of Biological and Health Sciences of Pontifical, Catholic University of Paraná (PUCPR), Street Imaculada Conceição, 1155, Curitiba 80215-901, Paraná, Brazil; rossanabaggio@gmail.com (R.B.S.); paulo_vicmar@hotmail.com (P.A.B.M.J.); michirabello@gmail.com (A.F.R.d.S.M.); guinaves95@gmail.com (G.N.); bergonsent@terra.com.br (N.B.N.); lnno.noronha@gmail.com (L.d.N.); julio.apfr@gmail.com (J.C.F.); guaritasouzalc@hotmail.com (L.C.G.-S.); 2 Department of Veterinary Medicine, Universidade Federal do Paraná (UFPR), Rua XV de Novembro, 1299, Curitiba 80060-000, Paraná, Brazil; Meilaalmeida41@gmail.com; 3 Advanced Therapy and Cellular Biotechnology in Regenerative Medicine Research Group, Pelé Pequeno Príncipe Research Institute & Pequeno Príncipe Faculties (FPP) Ave., Silva Jardim, 1632, Curitiba 80240-020, Paraná, Brazil; Dziedzic.dilceledz@gmail.com (D.S.M.D.); nadianr@gmail.com (N.N.d.R.); priscilaeferreira@gmail.com (P.E.F.S.); katherinecarv@gmail.com (K.A.T.d.C.)

**Keywords:** acellular amniotic membrane, tissue engineering, human umbilical cord mesenchymal stem cells, chondrocytes, tracheostomy

## Abstract

Acellular amniotic membrane (AM) has been studied, with promising results on the reconstruction of lesioned tissues, and has become an attractive approach for tracheal repair. This study aimed to evaluate the repair of the trachea with human umbilical cord mesenchymal stem cells (hucMSCs) differentiated in chondrocytes, grown on an experimental model. Tracheal defects were induced by surgical tracheostomy in 30 New Zealand rabbits, and the acellular amniotic membrane, with or without cells, was covering the defect. The hucMSCs were isolated and cultivated with chondrogenic differentiation over the culture of 14 days, and then grown on the AM. In this study, the AM was biocompatible and hucMSCs differentiated into chondrocytes. Our results demonstrated an important role for AM with cultured cells in the promotion of immature collagen, known to produce tissue regeneration. In addition, cartilaginous tissue was found at the tracheal defects, demonstrated by immunohistology results. This study suggests that this biomaterial implantation can be an effective future therapeutic alternative for patients with tracheal injury.

## 1. Introduction

Stenosis of the trachea is a major cause of morbidity and mortality after endotracheal intubation or tracheostomy [1]. Several deleterious effects are known to occur on the tracheal mucosa, including inflammation during trauma, granulation tissue formation, fibrosis, or scar formation [2].

The therapeutic options for tracheal defects are related to the length of the defect. It is known that the trachea can be reduced by half in adults and to by a third in small children being the gold standard treatment the end-to-end primary surgical anastomosis. When the defect exceeds these limits, it has been tried to replace the trachea using organic and artificial tubes that unfortunately causes severe complications such as infection, extrusion, obstruction, stenosis and chronic rejection [3].

Studies have suggested that the regeneration of the trachea through growth factors in platelet-rich plasma (PRP), such as platelet-derived growth factor (PDGF), and transforming growth factor-β (TGF-β), promotes tissue rejuvenation and tissue repair [4,5]. However, there are many obstacles to overcome, such as the rapid degradation of growth factors [6].

Finding the ideal material for tracheal replacement is still a challenge for regenerative medicine. The ideal material for tracheal replacement should present good biocompatibility and degradability, and have the ability of cell adhesion and proliferation [7]. For more than one century, the amniotic membrane has been used as an alternative in several areas of tissue engineering [8,9].

The amniotic membrane has growth factors and a biological structure that make it an ideal biomaterial, which contains epithelialization stimulation capacity, antibacterial properties, and defined mechanical strength. In addition, acellular amniotic membrane (AM) is the natural extracellular matrix which contains various growth bioactive factors that remain intrinsic after the decellularization process, such as VEGF, TGFb1, bFGF, and EGF. Through their bioinductive properties, the acellular membrane may also promote endogenous mechanisms of cell repair and regeneration, such as angiogenesis or vasculogenesis, that are typically present in the tissue [9].

In the field of cell therapeutic strategies, there are promising procedures that facilitate tracheal repair and regeneration. Similar to other stem cells, human umbilical cord mesenchymal stem cells (hucMSCs) are able to differentiate into local components of tissue injury, have low cost and immunogenicity, are minimally invasive, and with the ability to secrete cytokines and growth factors that help tissue regeneration [10]. This study aimed to assess the potential improvement of trachea function using hucMSCs differentiation on an acellular amniotic membrane for tissue repair in the tracheostomy model.

## 2. Materials and Methods

### 2.1. Animals

The experimental animal protocol of this study was approved by the Pontifical Catholic University of Paraná Animal Use Committee (approval No 01025-PUCPR). All rabbits were housed under standard conditions with food and water ad libitum on a 12-h day/night cycle (light on at 7 am) in individual cages, throughout the experimental period. All animal experiments were performed at the Experimental Laboratory of the Institute of Biological and Health Sciences at the Pontifical Catholic University of Paraná. The facility was structured for animal housing, as well as with an animal experimental surgical room for the proceedings. All experiments were performed according to the guidelines and the National Institutes of Health Guide for the Care and Use of Laboratory Animals [11].

### 2.2. Experimental Design

**Figure 1:**
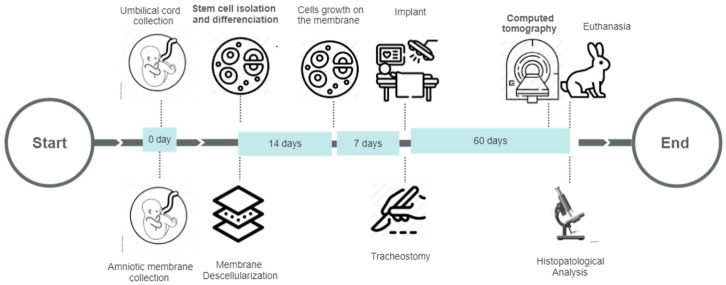
Experimental design: biomaterial collection, stem cell isolation and differentiation, membrane decellularization, tracheostomy, implant, computed tomography, euthanasia, and histopathological analysis.

Thirty New Zealand White rabbits weighing (3.15–3.44 kg) were used in this study. The animals were randomized into three groups: group C (*n* = 10), tracheostomy without treatment (control group); group AM (*n* = 10), tracheostomy with acellular amniotic membrane treatment, and group AM + hucMSCs (*n* = 10), tracheostomy with human umbilical cord mesenchymal stem cells (hucMSCs) differentiated in chondrocytes grown on an acellular amniotic membrane. The animals were subjected to tracheostomy and the membrane was implanted. Sixty days after surgery, they were analyzed by a micro-computed tomography scanner to assess trachea function. All rabbits were euthanized, and histopathological analysis was performed (Figure 1).

### 2.3. Isolation and Characterization of hucMSCs/Amniotic Membrane Decellularization

The amniotic membrane and human umbilical cords were obtained from full-term pregnant section surgery in Maternidade Victor Ferreira do Amaral Maternal and Child Health Hospital (Curitiba, Brazil). The patients were informed a priori and consented to donation. The hucMSC was obtained by tissue mass culture. Briefly, under sterile conditions, umbilical cord tissue was cut open, the surface membrane gently peeled, the umbilical vein and artery were removed, blood cleared from the tissue, and it was finally rinsed in saline.

For cell culture expansion, hucMSC were kept in basal medium (DMEM/F-12, 15% Hyclone FBS; U.S. Origin; GE, United States), 4% penicillin and streptomycin (Gibco; Invitrogen, Carlsbad, CA, USA), and 4% nonessential amino acids solution (Gibco; Thermo Fisher Scientific, Grand Island, NE, USA) at 5% CO2 and 37 °C. StemPro Chondrogenesis Differentiation Kit (Thermo Fisher Scientific, Grand Island, NE, USA), was used to differentiation cells. Differentiated cells were maintained for 2 weeks in culture [12].

Decellularization was performed with aseptic technique in a biological safety class II BioSAFE (Veco^®^). For this process, the membranes were removed from the pH 7.2 phosphate buffer (PBS) medium (Gibco) and treated with 0.01% SDS solution (sodium duodecyl sulfate) and SD (sodium deoxycholate) at 0.01% for 24 h at 37 °C, with the aid of a stirrer mechanic (Shaking Table 109 M, Nova Ética Ltd.a, Vargem Grande. Paulista, Brazil). Finally, the prepared AM was rewashed 3 times with PBS. Then preserved in PBS at 4 °C, according to the methodology described by Hopper Woodhouse, 2003 [13].

Afterwards, a fragment of the acellular amniotic membrane approximately 10 mm^2^ was cultured with approximately 3 × 10^5^ umbilical cord mesenchymal stem cells differentiation. Cell culture on the membrane decellularized was incubated in a 37 °C, 5% CO_2_ incubator for another 7 days.

### 2.4. Flow Cytometry Analysis

To verify the human umbilical cord’s mesenchymal stem cell origin, flow cytometric analysis was performed using the FACSCalibur system (BD Biosciences, San Jose, CA, USA). Immunophenotypic analyses for CD34, CD 45, CD105, CD 90, CD73 were performed with a commercially available kit (Stem Kit, Beckmann Coulter, Krefeld, Germany). An appropriate isotype-matched control antibody was used for all analyses.

### 2.5. Rabbit Tracheostomy Model and Membrane Implant

**Figure 2:**
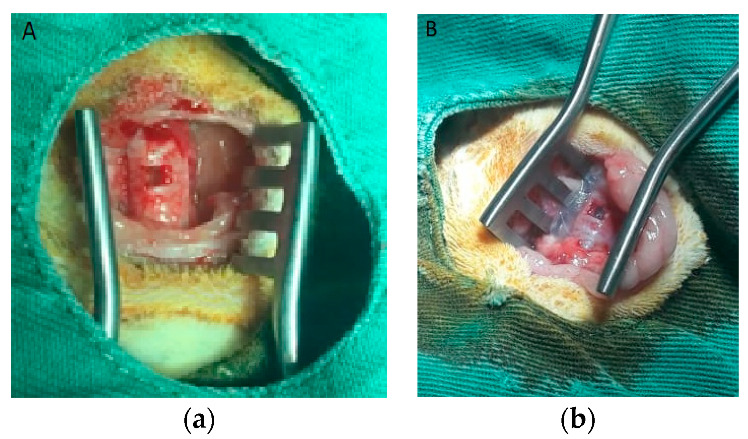
(**a**) tracheostomy procedure in New Zealand rabbit, trachea exposition with a 5 × 2 mm (10 mm^2^) defect made with a scalpel. (**b**) defect covered by a rectangular acellular amniotic membrane with human umbilical cord mesenchymal stem cells differentiated in chondrocytes.

The rabbit tracheostomy model was performed according to Jorge et al. [14]. The rectangular tracheal defect, of total thickness, 5 × 2 mm (10 mm^2^) was excised with a scalpel (Figure 2).

The animals were submitted to implantation on the tracheal defect. The suture was supported on four repair points at the ends of the rectangle with polypropylene thread 4.0 needled. After the end of the suture, the pre-tracheal tissues were approximated by continuous suture with needle 0 cotton thread. After implantation of the membrane cultivated with cells, the rabbits were clinically monitored and euthanized (8 weeks after implantation).

### 2.6. Computed Tomography Scanning Analysis

**Figure 3:**
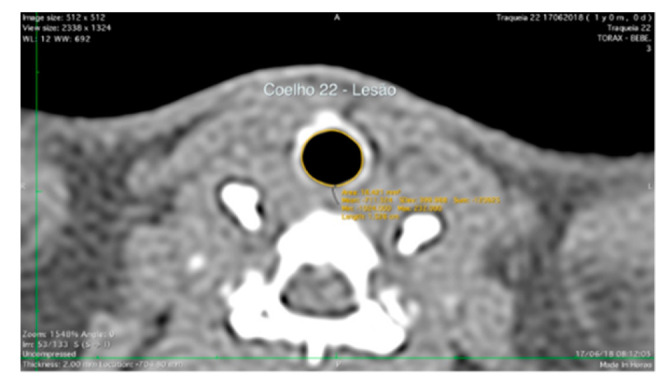
Post-contrast transverse computed tomographic images of the tracheal in a rabbit.

The scanned computed tomography images of each animal were taken at settings of 90 kV, 60 µA, and 280 ms, with a resolution of 512 × 512  in Digital Imaging and Communication in Medicine (DICOM) reader (OsiriX, version 6.5; Pixmeo, Bernex, Switzerland) in transverse and sagittal reformats (Figure 3). The lesion area was calculated from 2D reconstructed images using Horos Version 3.3 software (Geneva, Switzerland).

### 2.7. Euthanasia

All animals were euthanized with a lethal dose of pentobarbital sodium (thiopental) 100 to 250 mg/kg, injected intraperitoneally.

### 2.8. Histopathological Analysis

Sixty days post-operation, pieces of the tracheal rings were removed in defect sites and fixed in 10% formalin, dehydrated by different degree of ethanol, and embedded in paraffin. After the sectioning of samples (5-μm thickness), staining was done with H&E and Movat’s stains examined and collagen in trachea sections was stained using a picrosirius red kit, using quantitative polarized light microscopy for collagen alignment within the injury region under a microscope (Nikon Eclipse E400; Nikon, Tokyo, Japan), and evaluated with a digital image analysis system. In each one, the percentage of the area was calculated occupied by red and yellow (collagen I) and green (collagen III) fibers. Considering that the other types of collagen constitute very small fractions, for practical purposes, the sum of collagens I and III was considered as the total collagen of the defect. Five were analyzed in each section, ten fields with 100x magnification located on the defect line. To identify the chondrocyte cells, formalin-fixed and paraffin-embedded tissue sections were immunostained using the Vector^®^ M.O.M. Immuno-detection kit (Vector, Burlingame, CA, USA). The primary antibody was applied for 1 h at room temperature. Hyaline cartilage was detected by immunoperoxidase staining for an Anti-Aggrecan ARGxx mouse monoclonal (ab3773) (1:200; Abcam, Cambridge, UK), according to the manufacture instructions. The slides were then incubated with secondary biotin-labeled, affinity-isolated anti-mouse immunoglobulins (LSAB^®^ + Kit, Peroxidase; DAKO Corp, Carpinteria, CA, USA).

### 2.9. Statistical Analysis

Using one-way ANOVA and Kruskal–Wallis post hoc analysis, the Computed Tomography, tracheal average lumen area, collagen and histological. The normality of the variables was evaluated by the Shapiro–Wilk normality test. The Kruskal–Wallis exact test was used to compare qualitative variables between the experimental groups. *p* values < 0.05 were considered statistically significant. Data were analyzed with the software Graphpad Prism 9.1 software (Graphpad, San Diego, CA, USA).

## 3. Results

### 3.1. Cell Adhesion and Proliferation

**Figure 4:**
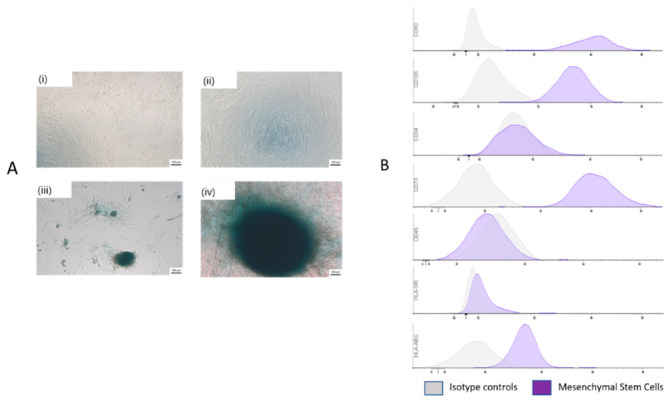
Morphology of characteristics of hucMSCs. (**A**) representative images of hucMSCs cells revealed cartilaginous characteristics with condensed hyaline matrices that were positive for Alcian blue at 7 days (i,ii) and 14 days (iii,iv). (**B**). Flow cytometry Histograms of Human umbilical Cord Mesenchymal Stem Cells. The histograms show that the population is positive for CD73, CD90, CD105 and HLA-ABC and negative for CD34, CD45, and HLA-DR.

After decellularization, the hucMSCs were cultured as described previously [14]. During in vitro culture, the monitoring of the cells demonstrated that they adhered very well to the amniotic membrane. Thus, after 14 days of monolayer outgrowth hucMSCs culture, the cells revealed cartilaginous characteristics with condensed hyaline matrices that were positive for Alcian blue staining (Figure 4A) and MSC surface markers CD73, CD90, and CD105 (≥95%) and little expression of the hematopoietic cell markers CD34, CD45, and HLA-DR (≤2%), markers were investigated by flow cytometry (Figure 4B). The results showed that the cells isolated from hucMSCs only include mesenchymal stem cells.

### 3.2. Computed Tomography Scanning Findings

The analysis with specialized image software provided quantitative data of the average transversal area of the lumen. After 60 days, the computed tomography analysis revealed that the average tracheal lumen area of the lesion region in the AM + hucMSCs group was increased compared with the AM group and the C group, although there was no significant result, with *p* = 0.211. Comparing the areas without injury to the groups, we did not observe any significance between them, demonstrating homogeneity between the groups, with *p* = 0.811 (Table 1).

### 3.3. Histological Analyses

The evaluation of the trachea showed a degree of stenosis, confirming the morphological and functional data.

**Figure 5:**
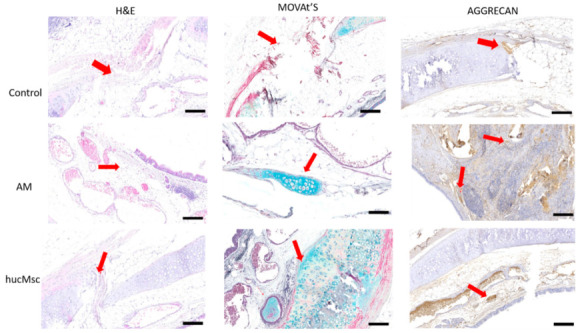
Histological images of grafts at 60 days after tracheal transplantation. Presence of Aggrecan in islands of immature cartilage on immunohistochemical staining (arrows) in AM and AM + hucMSCs group. The bar scale is 100 μm.

Histopathological analysis 60 d after surgery indicated the hucMSCs differentiation in cartilaginous tissue in AM + hucMSCs group, microscopic examination of tracheal path revealed the presence of immature cartilage islands, confirmed by H&E, Movat’s and Aggrecan which is typical of anti-aggrecan staining, adjacent to pseudostratified ciliated epithelium. In the AM group, cartilage was observed at the lesion area, in Movat’s stain (Figure 5).

### 3.4. Collagen Level Analysis

Collagen types I and III are the main collagen types of healthy organisms, and the percentage of collagen types I and III establish the evolution of the cellular repair.

**Figure 6:**
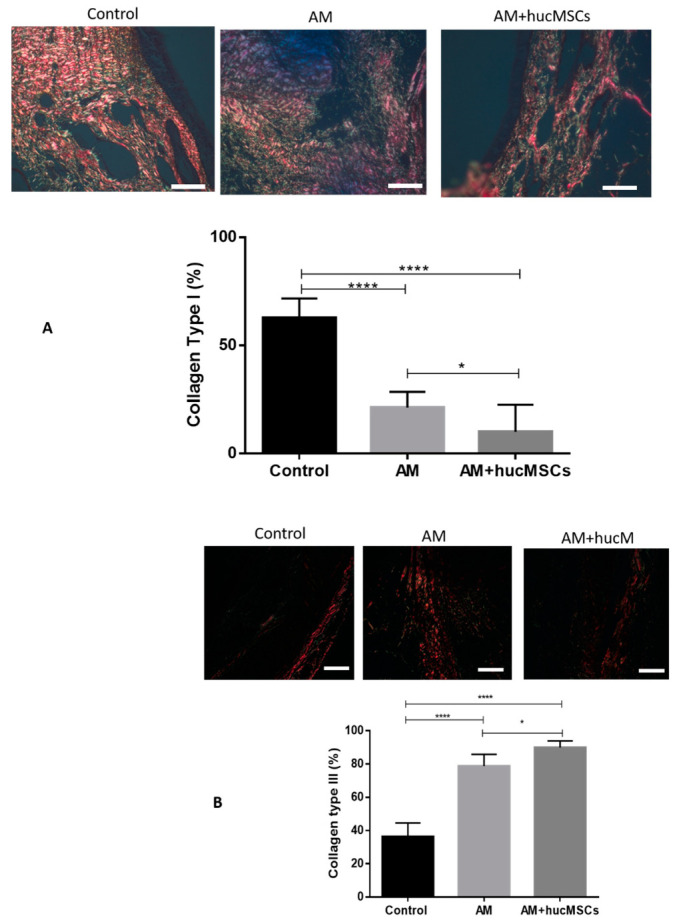
Images from Sirius red staining were obtained with circularly polarized light. (**A**,**B**) A quantitative analysis of ratio of collagen types I and III is shown in the corresponding histogram. Collagen type I was more predominant in control group. And collagen type III was more predominant in AM and AM + hucMSCs groups. Data are expressed as the mean ± SD (*n* = 10), **** stand for *p* < 0.0001, * stand for *p* < 0.05. Scale bars, 50 µm.

There is a significant increase in collagen III in the AM group and in the AM + hucMSCs group compared to group C, and a significant decrease in collagen I in the AM group and in the AM + hucMSCs group compared to group C with statistical significance (*p* < 0.0001). Results indicate a capacity of tissue regeneration, suggested by the development of immature collagen and formation of cartilage (Figure 6).

There is a significant difference when comparing group AM with group AM + hucMCSs in collagens I and III, with statistical significance (*p* < 0.05).

## 4. Discussion

The present results demonstrated the effectiveness of the acellular matrix that can contain the damage to the tracheal wall structure, especially to tracheal mucosa. Our findings indicate that an acellular matrix combined with cells differentiated in chondrocytes results in significantly reducing postoperative fibrosis formation after tracheal reconstruction.

A native trachea is a complex system that provides support to normal tissue and maintains cell–cell interactions, cell–matrix interactions, cellular differentiation, and tissue organization. Previous preclinical studies and clinical trials have used the amniotic membrane (AM) containing significant properties, such as effective anti-inflammatory proteins, antimicrobial effects, growth factors, proteases, and fibrosis inhibitors, being an attractive biomaterial in the field of regenerative medicine [15].

There are several types of methods, and cells have been proposed for replacing a defective trachea. However, an ideal material with more potential to tracheal replacement remain to be discovered [14].

The acellular human amniotic membrane (AHAM) offers an opportunity to cultivate cells and study tissue regeneration, as well as regulatory factors in a laboratory configuration. Decellularized AM has been successfully used as an attractive biomaterial for tissue repair and reconstruction in diverse animal studies and human applications [16,17].

The decellularization process is reported to preserve growth factors and other proteins that maintain the alignment of collagen fibers; thus, acellular amniotic membranes likely retain growth factors [5] and their structural strength [18]. Decellularization also improves biocompatibility, due to the absence of human epithelial cells that promote inflammatory responses. Our previous preliminary study was performed to examine the effects of this acellular matrix for tracheal reconstruction using the rabbit’s model, suggesting that AM can promote tracheal lumen repair through the in-migration of neighboring epithelial cells and chondrocytes [14].

Tracheoplasty was performed and the defects were replaced by the acellular matrix, combined with cells differentiated in chondrocytes. In the present study, it was demonstrated that the presence of the regeneration of cartilaginous tissue and pseudostratified ciliated epithet are crucial players in functioning respiratory and can promote the trachea regeneration as transplanted seed cells in the membrane at 2 months after surgery.

Thus, combining this therapy is a new insight for the treatment of tissue lesions and promoting tissue regeneration. Moreover, other studies have suggested the benefits of mesenchymal stem cell transplantation or seeded onto scaffolds to promote tracheal defects in rabbits [16,19].

Several studies have shown that human umbilical cord-derived mesenchymal stem cells (hucMSCs) have therapeutic benefits in tracheal cartilage repair and the early regeneration of epithelium tracheal [20,21,22,23].

The therapeutic benefit of AHAM can be obtained not only from decellularization, but also from a combination of components and growth factors inherent in the membrane, as well as its reepithelialization. New cartilage formation is one of the most important factors of tracheal regeneration. Researchers have suggested that growth factors in AHAM will promote tissue healing and tracheal repair [14].

The microCT lumen quantification data, although not significant, suggested an increase in the AM + hucMSCs group, as compared to the group that only received the acellular matrix, where they did not present significant transverse lumen areas, similar to native.

The results of this study showed that in the treatment groups, the regeneration effect on the treatment group was better than on the control group. The addition of acellular membrane (AM) improved tissue organization, while the group that received the differentiated hucMSCs showed better tissue repair, based on histology and collagen fiber dispersion. It was also observed a decrease in the size of the tracheal area, a reduction in the amount of collagen type I, and the presence of new chondrocytes and blood vessels in the tracheal fibrosis region of the AM + hucMSCs treated group.

Trachea treated with hucMSCs differentiated in chondrocytes exhibited collagen fiber dispersion range closer to the normal trachea, based on a quantitative analysis of stained samples of red picrosirius.

We assume, however, that the improvement in tissue repair may be due to the increased location of trachea mesenchymal stem cells at the site of the lesion, as well as the intrinsic growth factors of the acellular membrane and proliferation factors released by hucMSCs. Therefore, the acellular matrix combined with cells differentiated in chondrocytes can accelerate tracheal healing by promoting new cartilage formation.

This study was carried out in an experimental model. It will take considerable effort to implement this idea in clinical practices.

In conclusion, our results demonstrated an important role for the acellular matrix combined with cells in the promotion of collagen known to produce tissue regeneration and, thus, may present more effective future therapeutic results for patients suffering from tracheal injury.

## Figures and Tables

**Table 1:** Tracheal defect area: comparison between groups.

Tomography Analysis					
		Global Mean			
Variable	Group	Value (mm^2^)	Min; Max; Median (mm^2^)	*p* Value	Test
Lesion Area	C {*n* = 10)	18.71	11.37; 33.5; 13.1	0.211	ANOVA-Oneway
	AM {*n* = 10)	14.15	11.2; 16.1; 14.3		
	AM + hucMSCs {*n* = 10)	25.44	14.7; 47.4; 18.4		
Area without Lesion	C {*n* = 10)	25.10	19.1; 35; 24.2	0.811	Kruskal-Wallis
	AM {*n* = 10)	23.87	22.5; 26.2; 23.4		
	AM + hucMSCs {*n* = 10)	26.96	18.9; 47.9; 19.8		

## Data Availability

Not applicable.
